# Establishment of a CT-based radiomic feature robustness databank for OPC patients via image perturbation in a multi-institutional study: a practical method to safeguard model generalizability

**DOI:** 10.3389/fonc.2025.1464884

**Published:** 2025-11-24

**Authors:** Yongqiang Wang, Alex Zwanenburg, Jiang Zhang, Xinzhi Teng, Sai-Kit Lam, Jin Cao, Zongrui Ma, Ta Zhou, Yuanpeng Zhang, Hong Ge, Jing Cai

**Affiliations:** 1 Department of Health Technology and Informatics, The Hong Kong Polytechnic University, Hong Kong, Hong Kong SAR, China; 2 Department of Radiation Oncology, The Affiliated Cancer Hospital of Zhengzhou University & Henan Cancer Hospital, Zhengzhou, China; 3 National Center for Tumor Diseases Dresden/University Cancer Center (NCT/UCC), German Cancer Research Center Deutsches Krebsforschungszentrum (DKFZ), Heidelberg, Germany; 4 Faculty of Medicine and University Hospital Carl Gustav Carus, Technische Universität Dresden (TUD) Dresden University of Technology, Dresden, Germany; 5 Department of Radiation Physics, Helmholtz-Zentrum Dresden-Rossendorf (HZDR), Dresden, Germany; 6 OncoRay – National Center for Radiation Research in Oncology, Faculty of Medicine and University Hospital Carl Gustav Carus, Technische Universität Dresden (TUD) Dresden University of Technology, Helmholtz-Zentrum Dresden-Rossendorf, Dresden, Germany; 7 Department of Biomedical Engineering, The Hong Kong Polytechnic University, Hong Kong, Hong Kong SAR, China; 8 Research Institute for Smart Ageing, The Hong Kong Polytechnic University, Hong Kong, Hong Kong SAR, China; 9 The Hong Kong Polytechnic University Shenzhen Research Institute, Shenzhen, China

**Keywords:** radiomics, feature repeatability, model generalizability, oropharyngeal carcinoma, progression-free survival

## Abstract

**Purpose:**

To guide the preselection of highly repeatable radiomic features (RFs) in downstream analysis without further analysis its repeatability, a detailed radiomic feature robustness databank (RF-RobustDB) was established via image perturbation.

**Methods:**

Data on 1,274 oropharyngeal carcinoma (OPC) patients who had undergone pretreatment computed tomography (CT) imaging, collected from a public dataset. The original images and corresponding masks underwent systematic perturbations to simulate potential variations encountered during CT image rescanning, including translational shifts, rotational changes, random noise additions, and contour modifications. For each radiomic feature (RF), including unfiltered, wavelet-filtered, and Laplacian-of-Gaussian (LoG)-filtered features, we systematically quantified robustness against these perturbations by intraclass correlation coefficients (ICCs).

**Results:**

Out of 1395 first- and high-order RFs, 470 demonstrated excellent repeatability, i.e., a mean ICC of greater than 0.9. The use of these preselected highly repeatable RFs in model development improved the mean concordance (C) index in two external validation cohorts and reduced the mean C index gap between the training and external validation cohorts. These results demonstrate that the preselected high repeatable RFs from RF-RobustDB can effectively enhance radiomic model generalizability.

**Conclusions:**

The methodology employed to establish the RF-RobustDB is highly transferable to other tumor sites and different imaging modalities, which will facilitate the creation of RF-RobustDBs to guide the development of universally applicable radiomic models.

## 1 Introduction

The role and potential of radiomics in cancer management have been constantly expanding over the past decades, such as the distant metastases prediction of advanced nasopharyngeal carcinoma (NPC) (1), performing risk stratification of oropharyngeal cancer (OPC) (2), breast cancer risk estimation (3), and prediction of treatment response in non-small-cell lung cancer (NSCLC) (4). However, model generalizability remains the prime stumbling block for bend-to-bedside translation of radiomic models. To enhance the generalizability of radiomic models, concerted efforts have been made to enhance repeatability and reproducibility of radiomic features (RFs) for primary model generation (5–7). RF extraction, implemented prior to modeling process (8–11), is crucial for ensuring model reliability and generalizability. Although the Image Biomarker Standardization Initiative (IBSI) provides standardized guidelines for RF extraction (12), RF repeatability and reproducibility remain limited across institutions and imaging protocols (13–15). Consequently, these limitations represent fundamental challenges that need to be addressed before RFs can be effectively incorporated into modeling workflows.

Multiple variables influence the repeatability and reproducibility of RFs throughout the imaging process (13–27), such as scanner model (13, 14), scanner type (13, 15), scanning parameters (16), segmentation (17, 18), reconstruction (25), and preprocessing methods (26, 27). However, clear guidelines for selecting highly repeatable RFs in multi-institutional datasets remain unavailable. Test-retest methods pose additional challenges, as they may increase patients’ radiation exposure and consume medical resources unnecessarily. Manual re-segmentation further burdens radiologists with additional workload. Although phantom-based studies offer a radiation-free alternative for evaluating RF selection (28), their clinical applicability is limited due to imperfect simulation of human tissues. Given the practical constraints of test-retest studies and manual re-segmentation across institutions, there is an urgent need for a cost-effective, efficient, easily implementable, and clinically transferable RF robustness assessment method. Fortunately, A software-based image perturbation method proposed by Zwanenburg et al. offers a promising way to simulate the test-retest and re-segmentation process (29). This method simulates patient positioning during imaging, manual segmentation randomization, and varying noise levels of the imaging device. The effectiveness of image perturbation has been demonstrated by improved performance in radiomic models. For example, Teng et al. (30, 31) and Zhang et al. (6) applied image perturbation to select highly reproducible RFs that improved the reliability and generalizability of radiomic models. Moreover, image perturbation has been shown to achieve the same optimal reliability as test-retest imaging for constructing radiomic models (32).

Since the perturbation method demonstrates encouraging/promising capabilities in assessing feature stability, in this study, we aim to establish a reliable RF robustness databank (RF-RobustDB) via perturbation method for guiding the downstream development of radiomic models. Specifically, we included a large-scale of CT images of OPC patients from a total of 7 medical institution. The OPC dataset was obtained from the Cancer Imaging Archive (TCIA) (33). RFs from CT images with and without applications of popular imaging filters were analyzed. The repeatability of the RFs in the RF-RobustDB was quantified by one-way intra-class correlation coefficients (ICCs) (34). We adopted CT dataset for this study mainly owing to its wide-spreading popularity in the cancer management for pre-treatment planning, mid-treatment monitoring, and post-treatment evaluation, as well as the availability of dataset in the community.

Through systemic analysis, the cohort size effects on feature repeatability ensured that the sample size is sufficient to maintain the reliability of RF-RobustDB. Meanwhile, the RF-RobustDB-enhanced selection of highly repeatable RFs significantly improved the generalizability of progression-free survival (PFS) model. These results support the reliability of the comprehensive CT-based RF-RobustDB for OPC, offering a valuable insight into RF repeatability. Moreover, this study provided a comprehensive and generalized methodology for establishing an extensive RF-RobustDB applicable to diverse tissue sites and imaging modalities.

## 2 Materials and methods

### 2.1 Patient cohort

This retrospective study analyzed a dataset of pretreatment CT images from 1,418 head-and-neck cancer patients obtained from TCIA (33). The dataset included patients from seven medical institutions: 137 patients from the single-institution HEAD-NECK-RADIOMICS-HN1 (HN1) study (35, 36), 524 patients from the single-institution Radiomic Biomarkers in Oropharyngeal Carcinoma (RBOPC) study (37, 38), 298 patients from four institutions in the Head-Neck-PET-CT (HNPET) study (39, 40), and 459 patients from the single-institution Head and Neck Squamous Cell Carcinoma (HNSCC) study (41–43). To maintain consistency, only OPC patients with primary gross tumor volume (GTV) data were included, resulting in a final cohort of 1,274 OPC patients for establishing the site-specific RF-RobustDB. The baseline characteristics of the selected OPC patient are presented in **Table A1**. Due to the retrospective nature of this study, informed consent was not required.

**Table A1:** Baseline patient characteristics of the dataset in different cohorts.

Data cohort	Sex	Median age	Overall stage
HNSCC	Male: 395	57(28-87)	I-IV
	Female:64		
HN1	Male: 67	60(44-80)	I-IVb
	Female:21		
RBOPC	Male: 423	60(33-89)	I-IVb
	Female:101		
HNPET	Male: 151	63(34-90)	I-IVb
	Female:52		

### 2.2 Image perturbation

To simulate the inevitable variabilities in patient setup during image acquisition, a validated image perturbation method was used to mimic patient setup, randomized noise, and manual segmentation diversity. Translational and rotational perturbations were applied to the original (unfiltered) images and tumor masks to mimic patient position. Randomized noise was added to the original images to simulate noise variations during image acquisition. Contour randomization was applied to the tumor mask to mimic variations in manual tumor segmentation.

The image perturbation settings were based on previous studies on repeatability evaluation via image perturbation (6, 29, 31): translation distances were set to 0, 0.4, and 0.8 pixels; rotation angles were set to -20°, 0°, and 20°; noise levels were increased to 0, 1, 2, and 5 times the original noise level; and a three-dimensional random displacement field was used to deform segmented masks, resulting in randomized contours. For each voxel point, a random field vector component in each dimension is generated from a uniform distribution between -1 and 1. All z-components of the field vectors on the same slice are set to the same value to mimic the uniform inter-slice contour variations resulting from slice-by-slice contouring. The field vectors are then normalized in each dimension by the root mean square. Finally, they are smoothed using a Gaussian filter with a defined sigma value of 10 to ensure continuous changes in the random displacement field and to avoid sharp changes in the deformed contours. Sixty different perturbations were performed to enhance the reliability of our results, as previous studies have suggested that 40 different perturbations are sufficient (6, 30). During each perturbation operation, parameters from the four perturbation modes were randomly combined to simulate the uncertainty in variables during image rescanning.

### 2.3 RF extraction

Image pre-processing and RF extraction were conducted in accordance with the IBSI guidelines (12). Before RF extraction, all images were resampled to a 1 × 1 × 1 mm^3^ resolution, and re-segmentation was performed to limit pixel values between -150 and 180 HU, effectively excluding non-tumor tissue (such as air and bone) within the volume of interest (31). As a previous study suggested, using the fixed bin number between 8 and 128 discretize images can reduce the infinite possible number of intensity values to a finite set and image noise (44). Hence, a fixed bin number of 30 was used for image discretization in this study. RF extraction was performed using PyRadiomics v2.2 (45) in Python v3.7. Shape-based features, first-order features, and high-order features from the gray-level co-occurrence matrix (GLCM), gray-level run-length matrix (GLRLM), gray-level size-zone matrix (GLSZM), gray-level dependence matrix (GLDM), and neighboring gray-tone difference matrix (NGTDM) were extracted from the GTVs in original, Laplacian-of-Gaussian (LoG)-filtered (with sigma values of 1, 2, 3, 4, 5, and 6 mm) and Coiflet-1 wavelet-filtered images.

Fourteen shape-based features were extracted from each tumor mask, and 93 first-order and high-order features were extracted from each of the unfiltered, LoG-filtered, and wavelet-filtered images. Following image perturbation, we additionally extracted corresponding feature sets from all perturbed image variants. Finally, the RF-RobustDB contained 1,316 unfiltered, LoG-filtered, and wavelet-filtered RFs, and 78,960 perturbed features were extracted for ICC analysis.

### 2.4 RF repeatability assessment

Since the feature is extracted from different perturbated mask region, the assignment of perturbation parameters is independent to patients. Therefore, the robustness of each RF was quantified in terms of a one-way, random, absolute-agreement ICC, which was calculated using Equation 1, as follows (34).

(1) $$\begin{array}{c} ICC\left(1,1\right)=\frac{MS_{n}-MS_{W}}{MS_{n}+\left(k+1\right)MS_{W}} \end{array}$$

where $MS_{n}$ is the mean square for different patients, $MS_{W}$ is the mean square for residual sources of variance, and k is the number of perturbation times plus one for the unperturbed image. As recommended by a previous study (34), features with an ICC< 0.5 were regarded as having poor repeatability, ICC between 0.50 and 0.75 were regarded as having moderate repeatability, ICC between 0.75 and 0.90 were regarded as having good repeatability, and ICC > 0.9 were regarded as having excellent repeatability.

### 2.5 Establishment of the RF-RobustDB

To determine the reliable patient sample size (*n*) required for constructing the RF-RobustDB, the mean ICC values as a function of patient numbers were systematically analyzed. The methodology was implemented as follows: Starting with 10 patients, the sample size was incrementally increased by 10 patients up to 100, followed by 100-patient increments thereafter. For each specific patient-number subgroup, 10 rounds of random resampling were performed from the oropharyngeal carcinoma (OPC) datasets to calculate the corresponding mean ICC values. The six radiomic features (RFs) demonstrating the greatest variations in mean ICC values across different sample sizes were selected to illustrate the sample size dependency of ICC metrics. Based on this analysis, a patient number that showed a stabilized trend in mean ICCs was identified and ultimately used for establishing the RF-RobustDB.

### 2.6 RF selection and PFS model development

**Figure 1:**
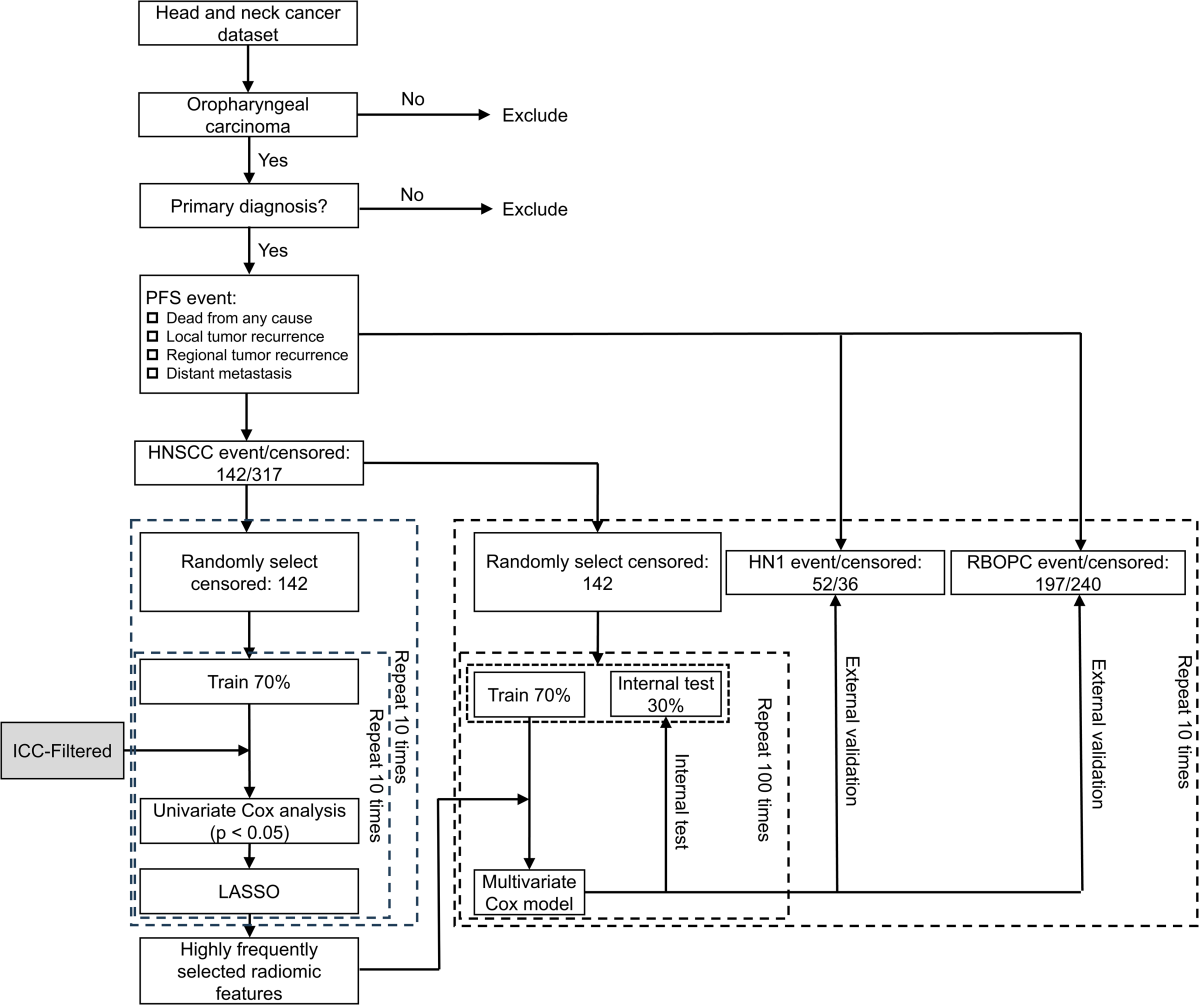
Workflow used for building progression-free survival (PFS) models based on CT images of oropharyngeal carcinoma patients. ICC = intraclass correlation coefficient; LASSO = least absolute shrinkage and selection operator; RF = radiomic feature; HNSCC = Head and Neck Squamous Cell Carcinoma; HN1 = HEAD-NECK-RADIOMICS-HN1; RBOPC = Radiomic Biomarkers in Oropharyngeal Carcinoma.

To demonstrate the efficacy of the RF-RobustDB, six PFS models were constructed using RFs selected through different ICC thresholds: (1) ICC > 0.9, (2) ICC > 0.85, (3) ICC > 0.8, (4) ICC > 0.75, (5) ICC > 0.5, and (6) non-preselected RFs. PFS events were defined as local/regional recurrence, distant metastasis, or death from any cause. **Figure 1** demonstrate the complete feature selection and modeling workflow. For the feature selection procedure, the highly reliable RFs derived from unperturbed images were initially chosen based on their robustness, as defined by the mean ICC. Subsequently, univariate Cox analysis was utilized to identify the RFs associated with PFS events within the pre-selected RFs in the training group. RFs with a *p*-value of less than 0.05 were considered significant. Finally, the least absolute shrinkage and selection operator algorithm was employed to select RFs with non-zero coefficients in the training group. The training groups were randomly bootstrapped 10 times from the 10 resampled balanced HNSCC dataset. The features that appeared frequently were selected to construct the PFS models.

To determine the optimal number of RFs for modeling, the relationship between the feature number and model performance was systematically investigated in the HNSCC dataset (**Figure A1**). The results revealed that the model constructed using non-preselected RFs exhibited optimal performance in the internal testing group when the feature number reached five (**Figure A1(A)**). Specifically, the mean C index exhibited the highest value in the testing cohorts, indicating the superior predictive ability of the model at this feature threshold. Moreover, using a greater number of RFs resulted in larger mean C index gaps between the training and internal testing groups. Similar trends of optimal performance were observed in the other five experiments (**Figure A1(B)-(F)**). Therefore, the top five RFs that most frequently appeared in all experiments were ultimately selected to ensure that the feature number would not introduce conflicts into the final results. The selected RFs for each experiment are listed in **Table A2**.

**Table A2:** Final selected radiomic features for each PFS model.

ICC threshold	Feature name
ICC>0	log-sigma-2-mm-3D_glszm_LowGrayLevelZoneEmphasis_30_binCount
	log-sigma-5-mm-3D_glszm_LowGrayLevelZoneEmphasis_30_binCount
	log-sigma-6-mm-3D_glszm_SmallAreaLowGrayLevelEmphasis_30_binCount
	wavelet-LLH_firstorder_Skewness_30_binCount
	log-sigma-3-mm-3D_glszm_ZoneEntropy_30_binCount
ICC>05	wavelet-HHH_firstorder_Energy_30_binCount
	wavelet-LHH_firstorder_Energy_30_binCount
	log-sigma-5-mm-3D_firstorder_Energy_30_binCount
	log-sigma-4-mm-3D_firstorder_Energy_30_binCount
	wavelet-LLH_firstorder_Energy_30_binCount
ICC>075	wavelet-LHH_glrlm_GrayLevelNonUniformityNormalized_30_binCount
	wavelet-LHH_glcm_Idn_30_binCount
	log-sigma-6-mm-3D_glcm_ClusterShade_30_binCount
	original_glcm_Correlation_30_binCount
	wavelet-LLL_glcm_JointAverage_30_binCount
ICC>08	wavelet-LHH_glcm_Idn_30_binCount
	log-sigma-6-mm-3D_glcm_ClusterShade_30_binCount
	log-sigma-5-mm-3D_glszm_ZoneEntropy_30_binCount
	original_glcm_Correlation_30_binCount
	log-sigma-4-mm-3D_glszm_SizeZoneNonUniformity_30_binCount
ICC>085	wavelet-LHH_gldm_SmallDependenceEmphasis_30_binCount
	wavelet-LLL_glrlm_ShortRunHighGrayLevelEmphasis_30_binCount
	wavelet-LLL_firstorder_Range_30_binCount
	log-sigma-2-mm-3D_firstorder_90Percentile_30_binCount
	log-sigma-6-mm-3D_glrlm_RunLengthNonUniformity_30_binCount
ICC>09	log-sigma-1-mm-3D_firstorder_Maximum_30_binCount
	log-sigma-2-mm-3D_glszm_SizeZoneNonUniformity_30_binCount
	original_glcm_Correlation_30_binCount
	log-sigma-3-mm-3D_glszm_SizeZoneNonUniformity_30_binCount
	log-sigma-6-mm-3D_glcm_DifferenceVariance_30_binCount

Multivariate Cox regression was employed to model the survival risks for PFS in the HNSCC dataset. The performance of the developed PFS models was evaluated by concordance (C) index for the training, internal testing, and external validation (HN1, RBOPC) cohorts. The HNPET dataset was excluded from external validation due to insufficient follow-up data on local/regional recurrence and distant metastasis. To assess the robustness of the models, the mean C index and its 95% confidence intervals were calculated in 100 bootstrap experiments on the 10 randomly resampled balanced datasets.

### 2.7 Model generalizability assessment

To assess model generalizability across external validation (EV) cohorts, a generalizability index (G) that quantifies the absolute difference in C index values between training and EV groups. The G index is defined by Equation 2:

(2) $$\begin{array}{c} G=\sum_{m}^{M}\frac{\left|C_{Train}-C_{EV_{m}}\right|}{M} \end{array}$$

where $C_{Train}$ represents the C index of the trained model, $C_{EV_{m}}$ is the C index for the *m*^th^ EV cohort, and *M* is the total number of EV cohorts. Lower G-index values indicate superior model generalizability, reflecting smaller performance discrepancies between training and validation datasets. The mean G index and corresponding 95% confidence intervals were calculated from 1,000 cross validation models to comprehensively evaluate the distribution of model generalizability performance.

## 3 Results

### 3.1 Patient-number dependence analysis

**Figure 2:**
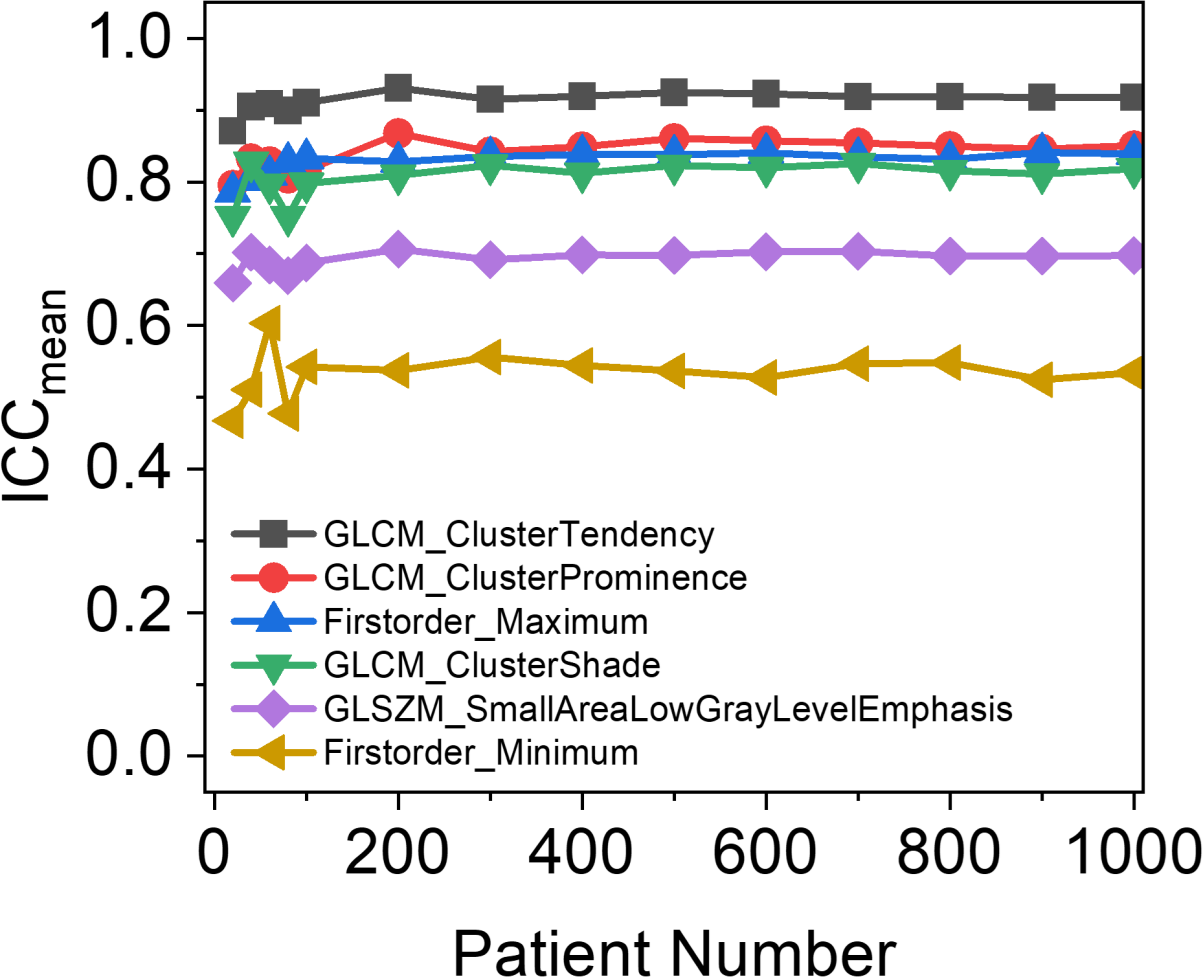
Patient-number dependence of mean intraclass correlation coefficients (ICCs) in six unfiltered radiomic features, which were selected based on the top six radiomic features with the largest variance in mean ICCs between different patient numbers.

**Figure 2** depicts the relationship between the number of patients and the mean ICCs of six RFs, which were selected as the top six RFs exhibiting the most significant variance changes with varying patient numbers. As the number of patients increased, the mean ICCs of the *Firstorder_Maximum* and *GLCM_ClusterTendency* features initially increased and then stabilized. In contrast, the mean ICCs of the remaining four selected features first fluctuated before eventually stabilizing. All six features tended to stabilize once the patient count reached 200. This stabilization trend of the mean ICCs demonstrates that the patient sample size used in this study was sufficient for constructing the RF-RobustDB. Specifically, 800 resampled patients were subjected to 100 iterations of resampling to compute the mean ICCs and their corresponding 95% confidence intervals.

### 3.2 Establishment and validation of the RF-RobustDB

#### 3.2.1 The repeatability of shape-based RFs

**Figure 3:**
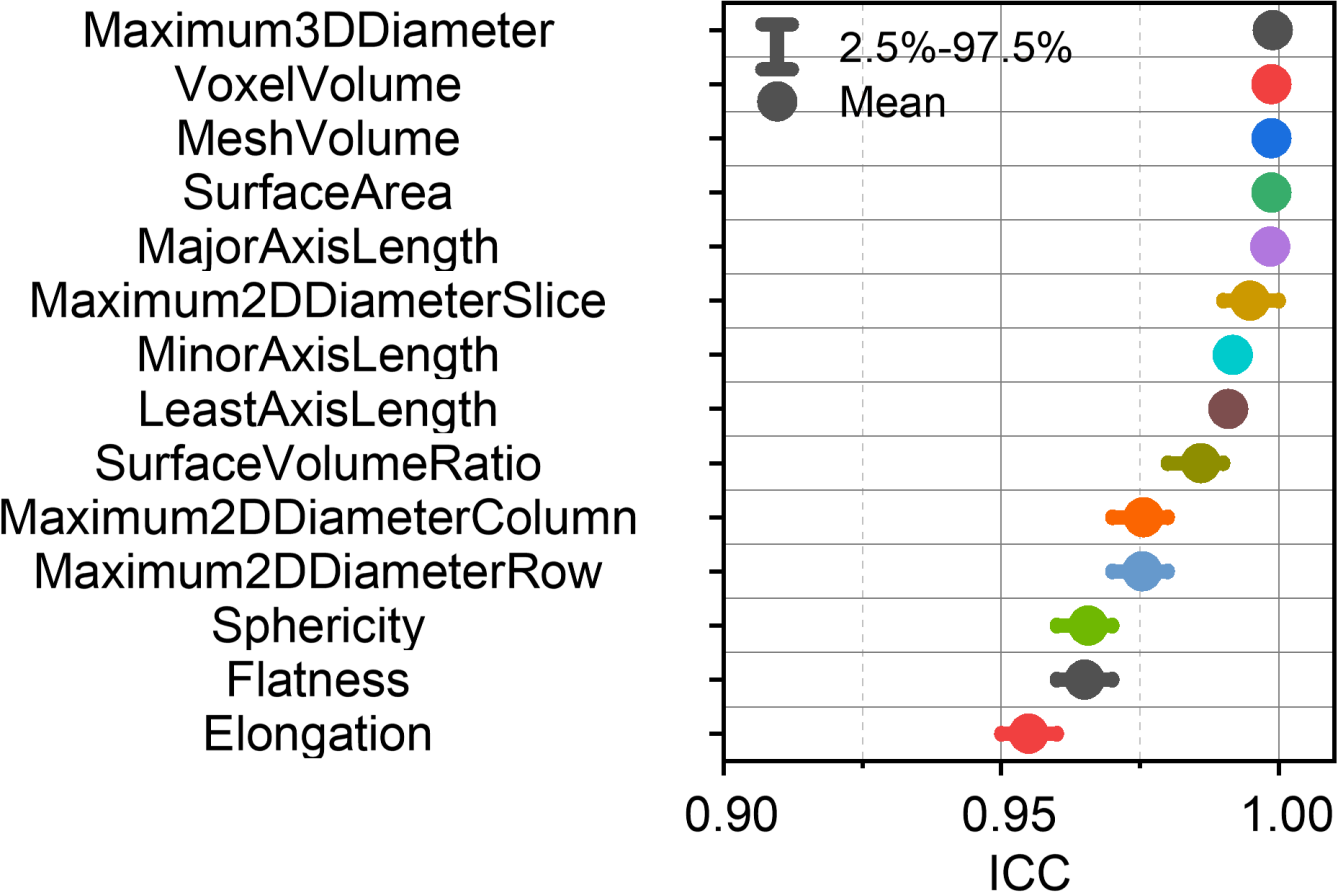
Mean intraclass correlation coefficients (ICCs) with 95% confidence intervals for each shape-based feature.

**Figure 3** presents a comprehensive evaluation of the ICCs for shape-based RFs across multiple simulated test conditions. Notably, all shape-based features demonstrated consistently high repeatability, with mean ICC values surpassing the 0.9 threshold (range: 0.955-0.999) across various perturbation scenarios. This robust performance supports the clinical applicability of shape-based RFs, as their measurements consistently reflect the tumor’s shape characteristics and are less influenced by other clinical factors.

#### 3.2.2 The repeatability of first-order and textural RFs

**Figure 4:**
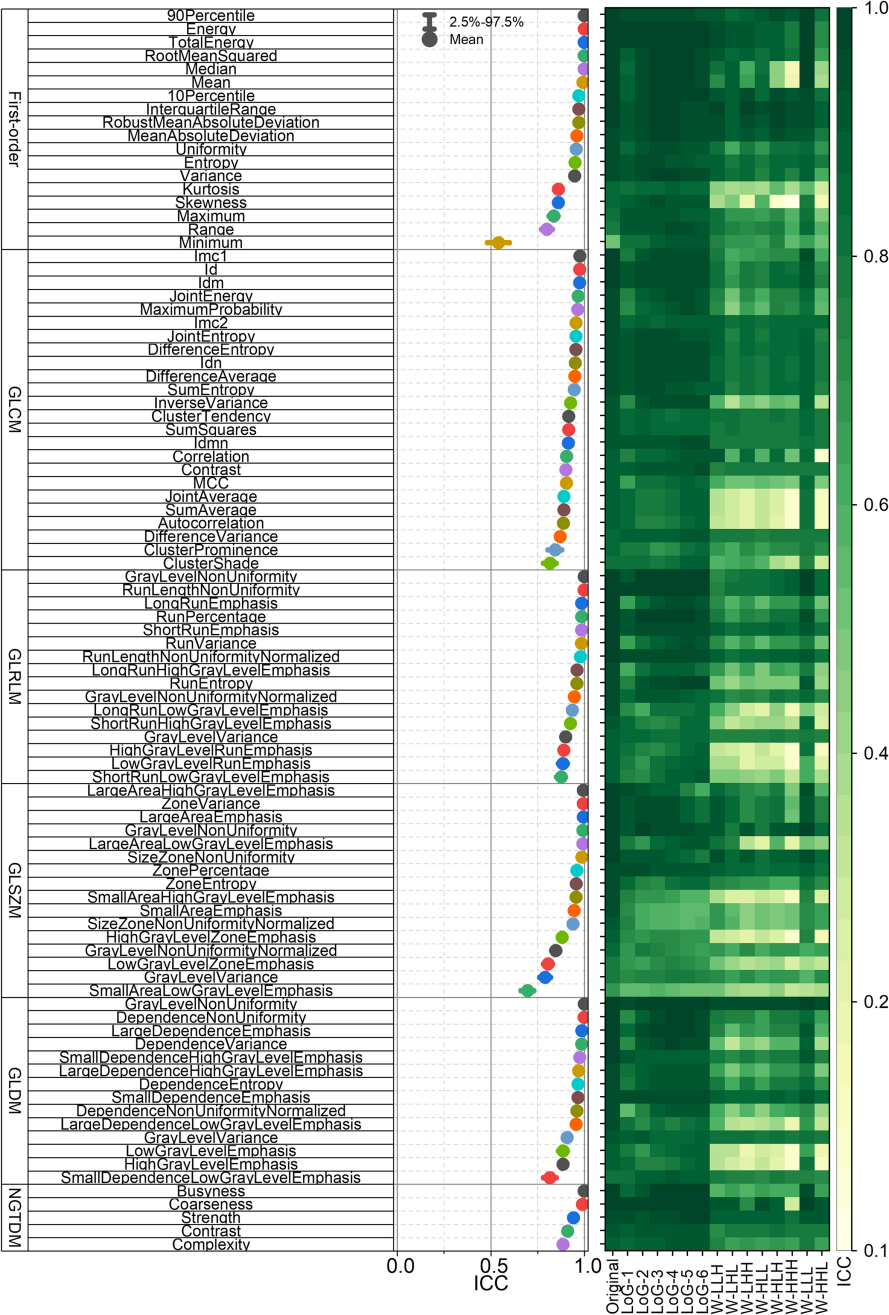
Intraclass correlation coefficients (ICCs) of first-order and textural radiomic features. GLCM = gray-level co-occurrence matrix; GLRLM = gray-level run-length matrix; GLSZM = gray-level size-zone matrix; GLDM = gray-level dependence matrix; NGTDM = neighboring gray-tone difference matrix.

**Figure 4** presents the ICCs of the first-order and textural RFs. The left panel of **Figure 4** displays the mean ICCs and their 95% confidence intervals for each unfiltered RF. The right panel of **Figure 4** gives the mean ICCs of the unfiltered, wavelet-filtered and LoG-filtered RFs. Collectively, **Figures 3** and **4** establish the RF-RobustDB. Detailed information on the mean ICCs and their 95% confidence intervals can be found in the Appendix.

#### 3.2.3 RF-RobustDB help improving the generalizability of PFS models

**Figure 5:**
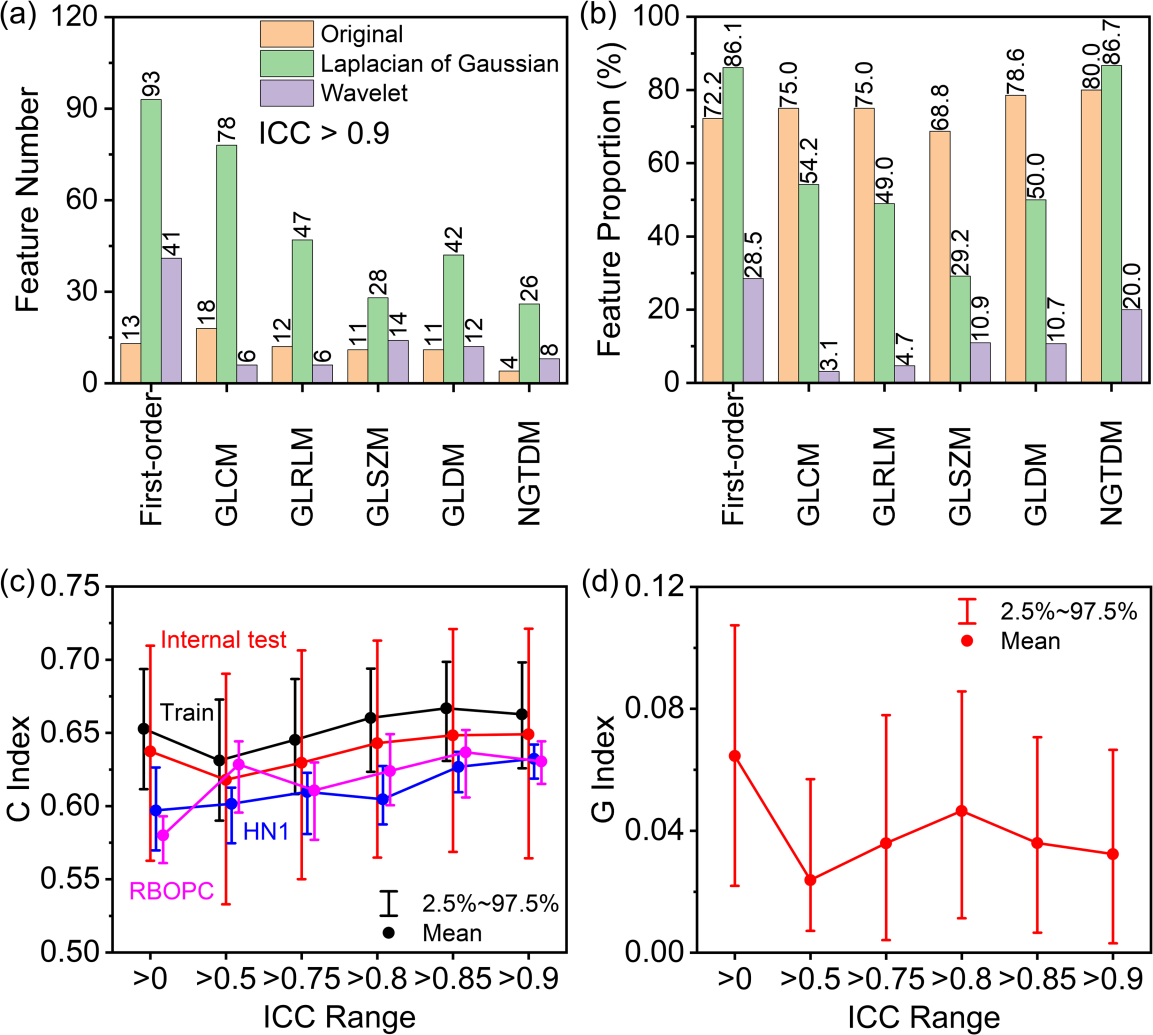
**(A)** Numbers and **(B)** proportions of excellent repeatable radiomic features in first-order, gray-level co-occurrence matrix (GLCM), gray-level run-length matrix (GLRLM), gray-level size-zone matrix (GLSZM), gray-level dependence matrix (GLDM), and neighboring gray-tone difference matrix (NGTDM) features selected based on mean ICCs. **(C)** Performance of progression-free survival (PFS) models built using preselected highly repeatable radiomic features with different mean intraclass correlation coefficient (ICC) thresholds in the training (Train), internal testing (Test), and two external validation (HN1 and RBOPC) cohorts. **(D)** Generalizability index **(G)** of the PFS models across the training and EV groups based on the concordance **(C)** index gap.

**Figure 5A, B** illustrates the selection of highly reproducible radiomic features (RFs) using the established RF-RobustDB. **Figure 5A** quantifies the absolute counts, while **Figure 5B** presents the relative proportions of these reproducible RFs across six feature classes: first-order, GLCM, GLRLM, GLSZM, GLDM, and NGTDM. Among 1,395 first-order and high-order RFs analyzed, 470 features (33.7%) demonstrated excellent repeatability, defined by mean ICCs > 0.9. Notably, unfiltered and LoG-filtered features exhibited significantly higher repeatability rates compared to wavelet-filtered features.

Using the established RF-RobustDB, six PFS models were systematically evaluated by recommended RFs at varying mean ICC thresholds. The C index in the training and internal testing groups first decreased and then increased as the ICC increased, as the red and black curve shown in **Figure 5C**. However, it is noteworthy that the EV cohorts (HN1 and RBOPC) demonstrated superior discriminative performance for models constructed using ICC preselected RFs, as shown by the pink and blue lines in **Figure 5C**. In addition, there was a smaller C index gap between the training and EV cohorts in the mean ICC preselected RF groups than in the non-preselected RF group, as shown by the G index trend in **Figure 5D**. The smallest mean G index was obtained in the RF groups preselected with a mean ICC of 0.5, and the largest mean G index was obtained in the non-preselected RFs groups. By taking into account the large C index and small G index of the RFs preselected with mean ICCs, it was found that the PFS models incorporating preselected RFs demonstrated significantly higher generalizability compared to those using non-preselected RFs.

## 4 Discussion

### 4.1 Result analysis

A robust RF-RobustDB of pretreatment CT-derived RFs in OPC patients was established through image perturbation. This database effectively guides the preselection of repeatable RFs and enhances the generalizability of multi-cohort PFS studies. A new G index was introduced to quantitatively evaluate the generalizability of the constructed PFS models. The methodology developed in this study can be easily extended to other anatomical sites and imaging modalities, providing a feasible solution for establishing standardized RF-RobustDBs to comprehensively assess RF repeatability across various clinical scenarios.

Our analysis of mean ICC dependence on patient cohort size (**Figure 2**) revealed that the six selected unfiltered RFs showing the highest variance across different sample sizes achieved stabilization when the patient number exceeded 200. This suggests that a minimum of 200 patients provides sufficient data for reliable ICC-based assessment of RF robustness, confirming that the sample size in this study (1,274) ensured the reliability of the RF-RobustDB. The RF-RobustDB evaluation demonstrated significant differences in feature repeatability between filtering methods: only 11.7% of wavelet-filtered RFs exhibited excellent repeatability (ICC>0.9), compared to 56.3% of Laplacian-of-Gaussian (LoG)-filtered RFs. This substantial disparity (44.6%) establishes the superior robustness of LoG-filtered features. The low repeatability of wavelet-filtered RFs likely stems from the characteristics of wavelet filtering, image resampling strategies, and perturbation settings (6). Therefore, radiomic model construction requires more stringent selection criteria for wavelet-filtered RFs compared to their LoG-filtered counterparts.

### 4.2 RF-RobustDB reliability analysis

The clinical utility of the RF-RobustDB was evaluated through external validation using two independent cohorts derived from separate institutions. Importantly, these validation cohorts were exclusively utilized for feature selection and model training phases, thereby maintaining the integrity of the validation process. Comparative analysis revealed that models incorporating RF-RobustDB-preselected features demonstrated superior performance in external validation, as evidenced by the higher mean concordance indices compared to models using non-preselected features and a reduction in the performance gap between training and validation cohorts. Zhang and colleagues improved the generalizability of a disease-free survival model for head and neck cancer by pre-selecting highly reproducible RFs using the perturbation method (6). The study by Gong et al. also provides compelling evidence supporting the critical importance of feature stability in radiomic analyses (46). Through systematic perturbation analysis of CT-derived imaging features in esophageal squamous cell carcinoma, their findings substantiate that incorporating high-stability features can enhance model performance in the external validation set. Thomas Louis et al. reported that robust features demonstrated superior predictive potential compared to non-robust features in predicting the outcomes of an external validation dataset (47). These studies collectively highlight the critical importance of feature stability for model generalizability, demonstrating findings consistent with our own results. This convergence of evidence further substantiates the fundamental value of establishing comprehensive RF-RobustDB to support subsequent radiomics research.

The reproducibility of RFs in clinical practice is subject to multiple influencing factors, such as patient positioning variability, segmentation quality, the noise level of medical imaging devices, and the variations between the performance of devices depending on their model and vendor, leading to differences in CT number values (13). Additionally, the reproducibility of RFs can be affected by scanning parameters and reconstruction algorithms (16, 25). This multifactorial variability explains the observed reduction reliability and generalizability when applying RFs in multi-cohort studies versus single-cohort studies. However, unlike variations in CT scanners or scanning modalities, variations in patient positioning, segmentation, and random noises are similar in various clinical circumstances, which allows perturbation methods to be a universal tool for assessing RF repeatability. Therefore, employing image perturbation across multiple institutions is a feasible approach for evaluating the robustness of RFs, as demonstrated by the enhanced generalizability of our PFS models. Considering these factors, the methodology employed in this study is both feasible and applicable for establishing RF-RobustDBs for other tumor sites and imaging modalities. The methodology is a promising approach for assessing the repeatability and enhancing the generalizability of radiomic models, thereby facilitating the development of more reliable and robust radiomic models with enhanced clinical translatability.

### 4.3 Existing limitations analysis

This study has several limitations that should be acknowledged. First, although our perturbation method simulated key variability sources including translations, rotations, random noise, and contour variations, they could not fully replicate all potential sources of variability encountered in clinical practice. For example, transient signal fluctuations that may occur during repeated scans under identical acquisition parameters cannot be effectively modeled. This inherent limitation underscores that image perturbation methods cannot entirely replace traditional test-retest validation approaches. Second, the optimal ICC thresholds for establishing reliable radiomic models remain controversial in the field. Additional investigations are required to establish evidence-based cutoff values for robust feature selection in clinical applications. Third, while our multi-institutional study design strengthened the generalizability of findings, the persistent effects of inter-scanner variability and acquisition parameter differences on feature reproducibility warrant further investigation. Furthermore, the dual use of our dataset for both RF-RobustDB construction and PFS model development may introduce circularity. Future validation should incorporate independent multi-institutional datasets to more rigorously assess the RF-RobustDB’s clinical utility. Addressing these limitations through continued research will be essential for optimizing the RF-RobustDB’s performance and expanding its applicability across diverse clinical implementations.

## 5 Conclusion

We have established a RF-RobustDB using an image perturbation approach for CT-derived RFs in OPC patients. The ICCs were calculated to quantify the reliability and repeatability of RFs. Through multi-cohort PFS experiments, we demonstrated the reference value of the RF-RobustDB, demonstrating that preselected highly repeatable RFs improved PFS model generalizability. To quantitatively assess model performance, we introduced a generalizability metric (G-index). The methodology we employed is cost-effective and easily applicable across different institutions, suggesting its potential extension to other lesion areas and imaging modalities. The comprehensive RF-RobustDB can facilitate robust RF selection when only small training datasets or single-institutional data are available, thereby enhancing the reliability, reproducibility, and generalizability of radiomic predictive models.

## Data Availability

The raw image date is available in the cancer image archive (https://www.cancerimagingarchive.net/collection/head-neck-radiomics-hn1/, https://www.cancerimagingarchive.net/collection/head-neck-pet-ct/, https://www.cancerimagingarchive.net/collection/hnscc/, https://www.cancerimagingarchive.net/collection/opc-radiomics/).
