# Supplementary material for: Establishment of a CT-based radiomic feature robustness databank for OPC patients via image perturbation in a multi-institutional study: a practical method to safeguard model generalizability
Source: Front Oncol. 2025 Nov 24;15:1464884. doi: 10.3389/fonc.2025.1464884 (PMC12683334; doi:10.3389/fonc.2025.1464884)
Supplement: Supplementary file 1 [file DataSheet1.docx]

***Supplementary Material***


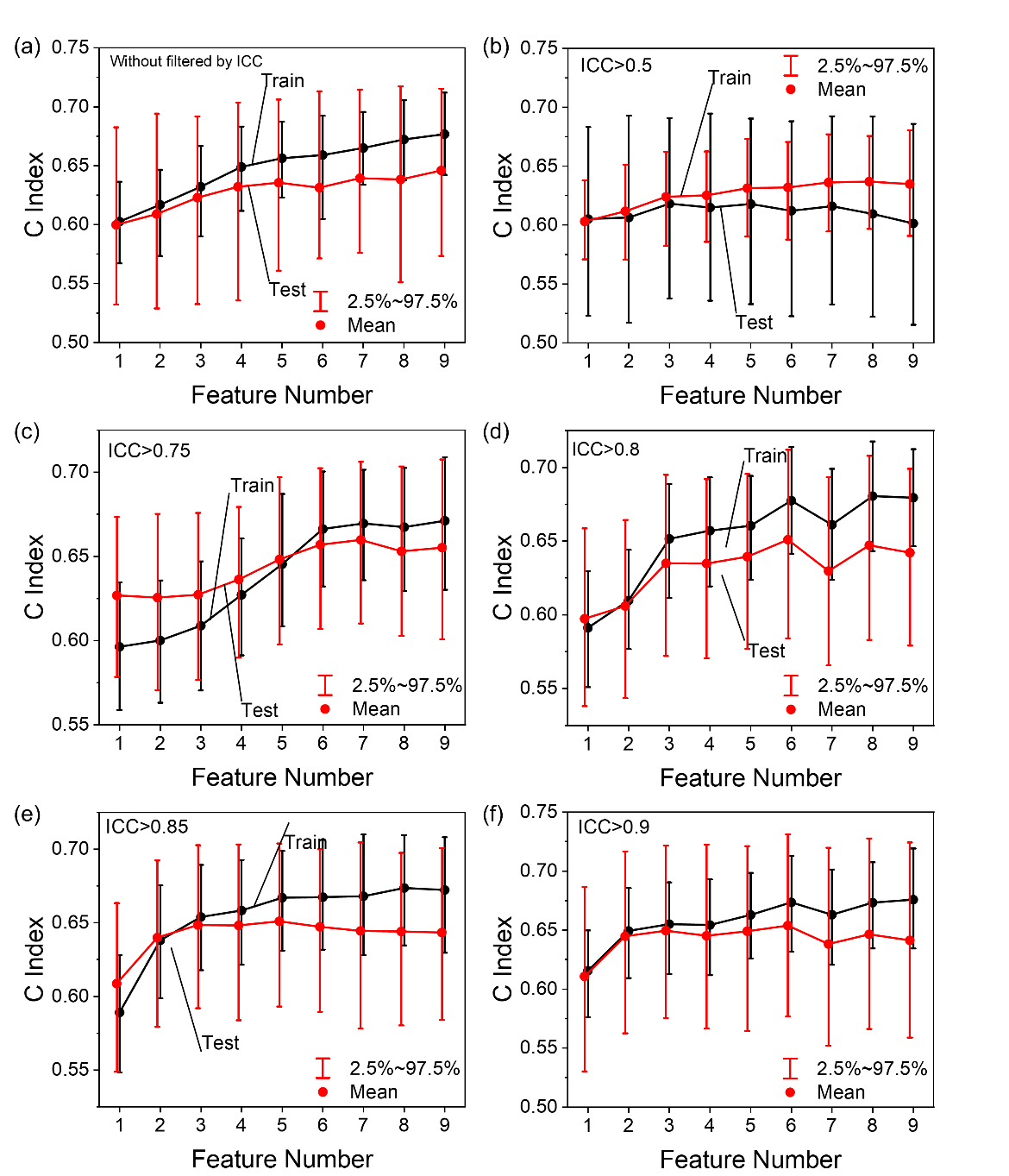


Figure A1. Feature number dependence of the performance of PFS models in the training and internal testing groups, where the PFS models were constructed by mean ICC coefficient thresholds larger than (a) zero, (b) 0.5, (c) 0.75, (d) 0.8, (e) 0.85, and (f) 0.9.
